# 

**Published:** 1886-12

**Authors:** 


					^edEMBER, 1886
THE
AMERICAN JOURNM
DENTAL SCIENCE.
EDITED BY
F. J. S. GORGAS, M. D., D. D. S.
UOL. 20
THIRD SERIES.
ftc\ 8.
BALTIMORE:
CNOWDEN & COWMAN.
LONDON:
TRUBNER & CO., 60 PATERNOSTER ROW.
PAYABLE IN ADVANCE.
PT1RT IS MFD MONTHLY.
$2.50 PER BNNUM.

				

